# IGF-1 release in the medial prefrontal cortex mediates the rapid and sustained antidepressant-like actions of ketamine

**DOI:** 10.1038/s41398-022-01943-9

**Published:** 2022-05-17

**Authors:** Satoshi Deyama, Makoto Kondo, Shoichi Shimada, Katsuyuki Kaneda

**Affiliations:** 1 Laboratory of Molecular Pharmacology, Institute of Medical, Pharmaceutical and Health Sciences, Kanazawa University, Kanazawa, 920-1192 Japan; 2 Department of Anatomy and Cell Biology, Graduate School of Medicine, Osaka City University, Osaka, 545-8585 Japan; 3 Department of Anatomy and Neuroscience, Graduate School of Medicine, Osaka Metropolitan University, Osaka, 545-8585 Japan; 4 Department of Neuroscience and Cell Biology, Graduate School of Medicine, Osaka University, Suita, Osaka 565-0871 Japan; 5 Addiction Research Unit, Osaka Psychiatric Research Center, Osaka Psychiatric Medical Center, Osaka, 541-8567 Japan

**Keywords:** Neuroscience, Pharmacology

## Abstract

Ketamine, an *N*-methyl-D-aspartate receptor antagonist, exerts rapid and sustained antidepressant actions. Preclinical studies demonstrated that the release of brain-derived neurotrophic factor (BDNF) and vascular endothelial growth factor in the medial prefrontal cortex (mPFC) is essential for the antidepressant-like effects of ketamine. However, the role of other neurotrophic factors in the antidepressant-like effects of ketamine has not been fully investigated. Since the intra-mPFC infusion of insulin-like growth factor 1 (IGF-1) reportedly produced antidepressant-like effects, the present study examined the role of endogenous intra-mPFC IGF-1 signaling in the antidepressant-like actions of ketamine. In vivo microdialysis showed that ketamine (10 and 30 mg/kg) significantly increased extracellular IGF-1 levels in the mPFC of male C57BL/6J mice for at least 5 h. Infusion of an IGF-1 neutralizing antibody (nAb; 160 ng/side) into the mPFC 15 min before or 2 h after ketamine injection blocked the antidepressant-like effects of ketamine in three different behavioral paradigms (forced swim, female urine sniffing, and novelty-suppressed feeding tests were conducted 1, 3 and 4 days post-ketamine, respectively). The ketamine-like antidepressant-like actions of the intra-mPFC infusion of BDNF (100 ng/side) and IGF-1 (50 ng/side) respectively were not blocked by co-infused IGF-1 nAb and BDNF nAb (200 ng/side). Moreover, intra-mPFC infusion of IGF-1 nAb 2 h post-ketamine blocked the antidepressant-like effects of ketamine in a murine lipopolysaccharide (LPS)-induced depression model. Intra-mPFC IGF-1 infusion also produced antidepressant-like effects in the LPS-challenged mice via mechanistic target of rapamycin complex 1 activation. These results suggest that persistent release of IGF-1, independently of BDNF, in the mPFC is essential for the antidepressant-like actions of ketamine.

## Introduction

Major depressive disorder (MDD) is a common mental illness affecting approximately 280 million people worldwide [1], causing serious individual and socioeconomic burden [2]. Depression is closely linked to suicide, and according to a World Health Organization report, around 800,000 people die from suicide annually [1]. However, current monoaminergic antidepressants, including selective serotonin reuptake inhibitors (SSRIs), produce a slow therapeutic response (weeks to months), and also have a limited efficacy: more than one-third of depressed patients fail to respond to multiple antidepressant treatments and are considered to experience treatment-resistant depression (TRD) [3, 4]. Therefore, there exists an urgent medical need for rapidly acting and more effective antidepressants with mechanisms different from those of conventional antidepressants.

Previous studies have shown that a single dose of ketamine, an open channel blocker of *N*-methyl-D-aspartate (NMDA) receptor, exerts rapid (within hours) and sustained (up to 7 days) antidepressant effects even in patients with TRD [5, 6] and similar antidepressant-like behavioral effects in rodents [7–9]. Although the cellular and molecular mechanisms underlying the unique antidepressant effects of ketamine remain elusive, preclinical studies have demonstrated that the antidepressant-like actions of ketamine require the release of brain-derived neurotrophic factor (BDNF) and vascular endothelial growth factor and activation of downstream mechanistic target of rapamycin complex 1 (mTORC1) signaling in the medial prefrontal cortex (mPFC) [7–13]. However, it is still unknown whether other neurotrophic factors are also involved in the antidepressant effects of ketamine.

Insulin-like growth factor 1 (IGF-1) is a neurotrophic factor that plays important role in neuronal development and various functions in the brain [14]. IGF-1 has been reported to be involved in antidepressant effects. Previous studies have shown that systemic and central administration of IGF-1 produces sustained (at least 6 days) antidepressant-like behavioral effects in various rodent models of depression [15–19]. In addition, it has been reported that IGF-1 administration into the mPFC produced antidepressant-like effects in rodents [15]. However, the possible role of endogenous IGF-1 in the antidepressant-like actions of ketamine remains unknown. Thus, in this study, we examined whether IGF-1 signaling in the mPFC mediates the antidepressant-like actions of ketamine.

## Materials and methods

### Animals

Male C57BL/6J mice (7–10 weeks, *n* = 214) were either purchased from Japan SLC (Hamamatsu, Japan) or bred in the animal facilities of Kanazawa University, Osaka City University, and Osaka University. They were group-housed and maintained at a constant ambient temperature (22 ± 2 °C) under a 12 h light/dark cycle with food and water available ad libitum. Animal use and procedures were approved by the Institutional Animal Care and Use Committees of Kanazawa University, Osaka City University, and Osaka University, and all efforts were made to minimize suffering of mice. Mice were randomly allocated into each treatment group and the investigators were not blinded during group allocation.

### Reagents

Ketamine (Ketalar, Daiichi-Sankyo, Tokyo, Japan) was diluted with sterile saline. A goat anti-mouse IGF-1 neutralizing antibody (nAb; Cat No. AF794), normal goat immunoglobulin G (IgG), normal sheep IgG, and recombinant mouse IGF-1 were obtained from R&D Systems (Minneapolis, MN) and reconstituted according to the manufacturer’s instructions. A sheep anti-BDNF nAb (Cat No. AB1513P, Millipore, Billerica, MA) was also reconstituted according to the manufacturer’s instructions. Recombinant BDNF was provided by Sumitomo Dainippon Pharma Co. Ltd. (Osaka, Japan) and dissolved in sterile phosphate-buffered saline (PBS) containing 0.1% bovine serum albumin (BSA; Wako, Osaka, Japan). Rapamycin (an mTORC1 inhibitor; LC laboratories, Woburn, MA) was dissolved initially in DMSO and then diluted with sterile PBS containing BSA (final concentrations of DMSO and BSA were 10 and 0.09%, respectively). Lipopolysaccharide (LPS, serotype 0127:B8; Sigma-Aldrich, St. Louis, MO) was dissolved in sterile saline.

### In vivo microdialysis

In vivo microdialysis was conducted as previously described [20]. Under sodium pentobarbital anesthesia (60 mg/kg, i.p.), a microdialysis guide cannula (PEG-4; Eicom, Kyoto, Japan) was placed in the mPFC (1.8 mm rostral, 0.4 mm lateral, 0.8 mm ventral to bregma) and fixed to the skull. After surgery, mice were allowed to recover for at least 2 days. Before the experiment, a microdialysis probe (PEP-4-2, membrane length 2 mm; Eicom) was inserted through the guide cannula. To obtain stable baseline samples, the probe was perfused with Ringer’s solution (147 mM NaCl, 4.0 mM KCl, 2.3 mM CaCl_2_) containing 0.15% BSA (Wako) in a push-pull manner using a micropump at 10 µL/min for at least 3 h before baseline sample collection [20]. Then, the probe was perfused at 1 µL/min, and eight 60 min dialysates were collected and the first two samples were taken as baseline samples. Immediately after collection of the second baseline sample, each mouse was administered intraperitoneally (i.p.) with either saline or ketamine (3, 10, or 30 mg/kg). After the experiment, the probe position was histologically verified. IGF-1 content in dialysate samples was analyzed using the Quantikine mouse/rat IGF1 immunoassay ELISA kit (R&D Systems), according to the manufacturer’s instructions. Data were calculated as percentage change from the basal dialysate concentrations, with 100% defined as the average of two samples before drug administration. Mouse blood samples were collected 3 h after i.p. injection of either saline or ketamine (10 mg/kg). Blood samples were allowed to clot for 2 h at room temperature and centrifuged for 20 min at 2000 × *g*. The serum samples were assayed using the Quantikine mouse/rat IGF1 immunoassay ELISA kit, as described above.

### Surgery and drug treatments

Intra-mPFC infusions were performed as previously described [12, 21, 22]. Briefly, mice were anesthetized with chloral hydrate (400 mg/kg, i.p.) and implanted with a bilateral 26-gauge guide cannula (Plastics One, Roanoke, VA) above the infusion sites in the mPFC (1.8 mm rostral, ±0.4 mm lateral, 2.5 mm ventral to bregma). After surgery, mice were housed individually and allowed to recover for at least 6 days. The mPFC infusions were done in conscious and freely moving mice. Mice were bilaterally infused with an IGF-1 nAb (160 ng/side), control goat IgG (160 ng/side), BDNF nAb (200 ng/side), control sheep IgG (200 ng/side), BDNF (100 ng/side), IGF-1 (50 ng/side), rapamycin (0.01 nmol/side), or appropriate vehicle into the mPFC (0.2 µL/side; 0.2 µL/min) using a bilateral 33-gauge injector (Plastics One) that protruded 0.3 mm beyond the tip of the guide cannula. The injector was kept in place for an additional 1 min to allow for diffusion. Ketamine (10 mg/kg) was administered i.p.

### Forced swim test (FST)

The forced swim test was conducted as previously described [12, 21, 22]. Each mouse was placed in a 4 L beaker containing water (24 ± 1 °C, 15 cm depth) and the immobility time was measured between 2 and 6 min in a blinded manner.

### Locomotor activity test

Locomotor activity (LMA) test was performed as previously described [22]. Each mouse was placed in a testing chamber (L38 × W26 × H24 cm) for 10 min and the total distance traveled was monitored using Smart 3.0 software (Panlab Harvard Apparatus, Holliston, MA).

### Female urine sniffing test (FUST)

FUST was conducted as previously described [12, 21, 22]. The FUST is based on the attraction of male rodents to pleasurable pheromones in female urine and serves as a measure of reward-seeking behavior [23]. In this test, mice were exposed to a cotton-tipped applicator infused with fresh urine from females of the same strain for 5 min. The time spent sniffing the cotton-tipped applicator was measured in a blinded manner. Estrous cycle was not monitored because previous studies have reported no influence of the cycle on male investigations [24, 25].

### Novelty-suppressed feeding (NSF) test

NSF test was carried out as previously described [12, 21]. Mice were food-deprived overnight and placed in an open field with a small amount of food in the center. The latency to feed was measured with a cut-off time of 15 min in a blinded manner. After the NSF, home cage feeding (HCF) during a 10 min period was measured to verify motivation to feed.

### Tail suspension test (TST)

TST were conducted as previously described [12]. Each mouse was suspended with its tail fixed to a hook by a small piece of adhesive tape. The immobility time was measured for 6 min in a blinded manner.

### LPS-induced depression model

LPS (0.8 mg/kg) or saline was i.p. injected into each mouse. This LPS dose produces depression-like behaviors 1 day after an LPS challenge without affecting LMA [26–28]. The LMA test, TST, and FST were conducted 24, 26, and 28 h, respectively, after the LPS challenge.

### Histology

After behavioral tests, histological analyses were conducted. Coronal sections (50 µm) were prepared on a cryostat and stained with thionin to confirm the infusion sites. Animals with incorrected infusion placements (*n* = 14) were excluded from analyses.

### Statistical analyses

Sample sizes were determined based on similar studies which are sufficient to obtain statistical significances, although no statistical methods were used to predetermine sample sizes. Data are presented as means ± SEM. The data were analyzed by one-way or two-way analysis of variance (ANOVA) followed by the Holm–Sidak’s or Tukey’s post hoc test using GraphPad Prism 6 or 9 (GraphPad Software, La Jolla, CA). In vivo microdialysis data were analyzed by two-way repeated-measures ANOVA followed by the Holm–Sidak’s post hoc test. The data of serum IGF levels were analyzed by two-tailed *t*-test. Differences with *p* < 0.05 were considered significant.

## Results

### Ketamine induces IGF-1 release in the mPFC

**Fig. 1:**
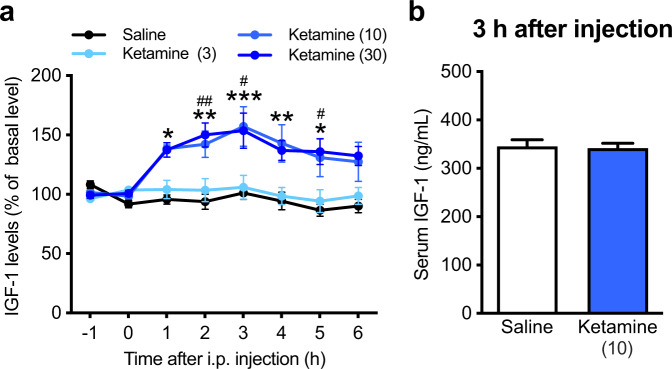
Ketamine induces IGF-1 release in the mPFC. **a** IGF-1 levels in extracellular fluid in the mPFC after i.p. injection of either saline (*n* = 13) or ketamine (3 mg/kg, *n* = 6; 10 mg/kg, *n* = 13; 30 mg/kg, *n* = 6; treatment × time interaction, *F*_21,238_ = 2.796, *p* < 0.0001). *p < 0.05, **p < 0.01, ***p < 0.001, saline vs. ketamine 10 mg/kg; ^#^*p* < 0.05, ^##^*p* < 0.01, saline vs. ketamine 30 mg/kg (two-way repeated measures ANOVA followed by Holm–Sidak’s post hoc test). **b** Serum IGF-1 levels 3 h after i.p. injection of either saline (*n* = 7) or ketamine (10 mg/kg; *n* = 7; *t*_12_ = 0.219, *p* = 0.8304, two-tailed *t*-test). Data are expressed as means ± SEM.

To study the possible relationship between ketamine and IGF-1, we first examined IGF-1 levels of extracellular fluid from the mouse mPFC after i.p. injection of ketamine (3, 10, or 30 mg/kg) by in vivo microdialysis. The baseline concentration of IGF-1 was 89.72 ± 8.17 pg/mL (*n* = 38). Ketamine (10 mg/kg) significantly increased extracellular IGF-1 levels in the mPFC for at least 5 h. Higher dose (30 mg/kg) increased IGF-1 levels in the mPFC to a similar extent as those seen following injection of 10 mg/kg, while lower dose (3 mg/kg) failed to significantly increase IGF-1 levels compared with saline control (Fig. 1a). Meanwhile, serum IGF-1 levels remained unchanged after ketamine injection (10 mg/kg; Fig. 1b). These results suggest that ketamine induces persistent IGF-1 release in the mPFC.

### Intra-mPFC infusion of IGF-1 nAb at 15 min pre- and 2 h post-ketamine blocks the rapid and sustained antidepressant-like effects of ketamine

**Fig. 2:**
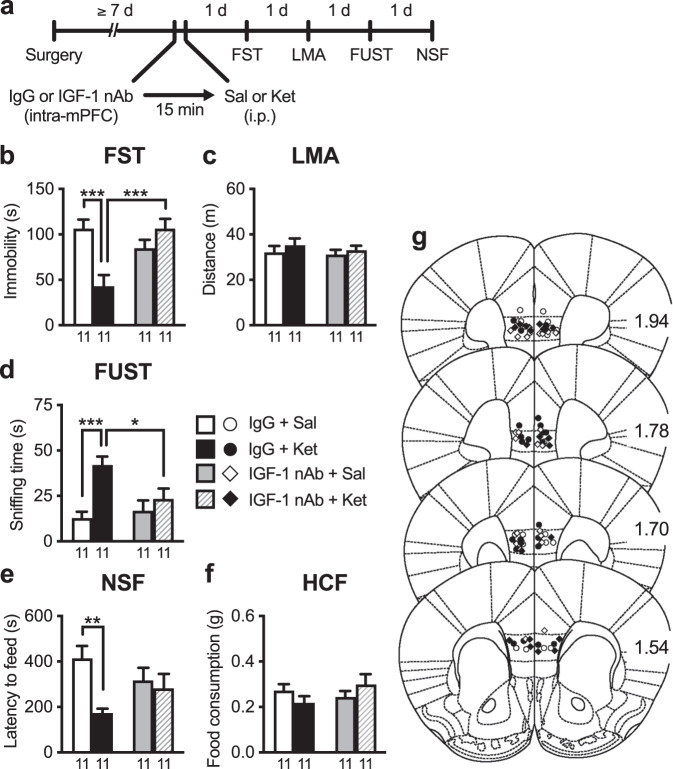
Intra-mPFC infusion of IGF-1 nAb 15 min prior to ketamine blocks the antidepressant-like effects of ketamine. **a** Experimental timeline for behavioral testing after intra-mPFC infusion of either control IgG (160 ng/side) or IGF-1 nAb (160 ng/side) and i.p. injection of either saline or ketamine (10 mg/kg). **b** Immobility time in the forced swim test (FST) 1 day after intra-mPFC and i.p. injections (interaction, *F*_1,40_ = 15.7, *p* = 0.0003, *n* = 11). **c** Locomotor activity (LMA) 2 days after intra-mPFC and i.p. injections (interaction, *F*_1,40_ = 0.0610, *p* = 0.806, *n* = 11). **d** Time spent sniffing female urine in the female urine sniffing test (FUST) 3 days after intra-mPFC and i.p. injections (interaction, *F*_1,40_ = 5.36, *p* = 0.0259, *n* = 11). **e** Latency to feed in the novelty-suppressed feeding (NSF) test 4 days after intra-mPFC and i.p. injections (interaction, *F*_1,40_ = 4.18, *p* = 0.0475, *n* = 11). **f** Home cage feeding (HCF) just after the NSF (interaction, *F*_1,40_ = 0.281, *p* = 0.101, *n* = 11). **g** Schematic representation of mPFC infusion sites. Plates are from ref. [50]; 1.94, 1.78, 1.70, and 1.54 indicate distances (mm) from bregma. Data are expressed as means ± SEM. *p < 0.05, **p < 0.01, ***p < 0.001 (two-way ANOVA followed by Holm–Sidak’s post hoc test).

To examine the role of IGF-1 release in the mPFC in the antidepressant-like behavioral effects of ketamine (10 mg/kg, i.p.), mice were infused with either an IGF-1 nAb (160 ng/side) or control IgG (160 ng/side) into the bilateral mPFC 15 min before i.p. injection of ketamine, and were subjected to the FST, LMA test, FUST, and NSF test (Fig. 2a). In control IgG-infused mice, ketamine significantly decreased immobility in the FST (Fig. 2b), increased sniffing of female urine in the FUST (Fig. 2d), and decreased latency to feed in the NSF test (Fig. 2e). However, these antidepressant-like effects of ketamine were completely blocked in IGF-1 nAb-infused mice (Fig. 2b, d, e). These treatments had no effect on LMA and HCF (Fig. 2c, f), and intra-mPFC infusion of IGF-1 nAb had no significant effects on any of the behaviors tested in saline-injected mice (Fig. 2b–f). These results suggest that IGF-1 release in the mPFC is required for the induction of the rapid and sustained antidepressant-like actions of ketamine.

**Fig. 3:**
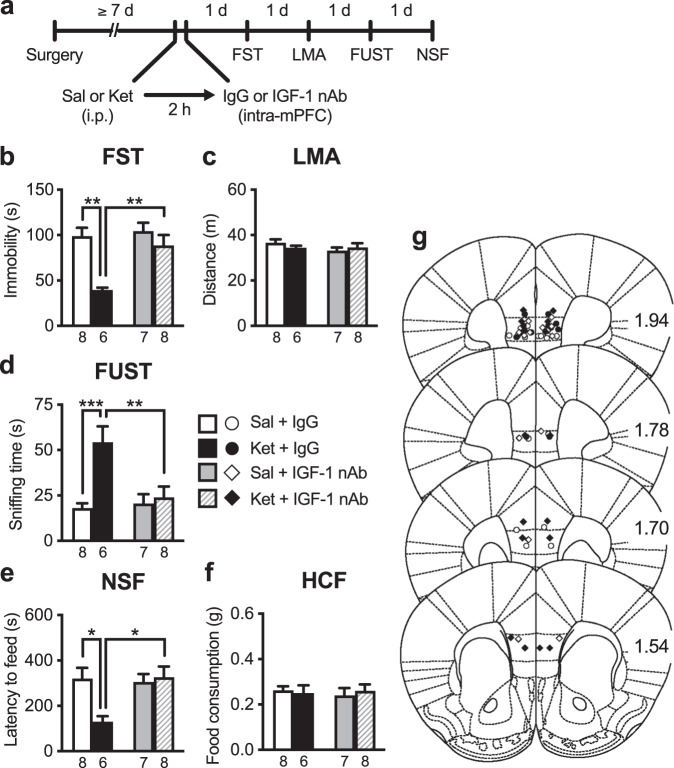
Intra-mPFC infusion of IGF-1 nAb at 2-h post-ketamine blocks the antidepressant-like effects of ketamine. **a** Experimental timeline for behavioral testing after i.p. injection of either saline or ketamine (10 mg/kg) and intra-mPFC infusion of either control IgG (160 ng/side) or IGF-1 nAb (160 ng/side). **b** Immobility time in the forced swim test (FST) 1 day after i.p. and intra-mPFC injections (interaction, *F*_1,25_ = 5.24, *p* = 0.0308, *n* = 6–8). **c** Locomotor activity (LMA) 2 days after i.p. and intra-mPFC injections (interaction, *F*_1,25_ = 1.22, *p* = 0.279, *n* = 6–8). **d** Time spent sniffing female urine in the female urine sniffing test (FUST) 3 days after i.p. and intra-mPFC injections (interaction, *F*_1,25_ = 8.59, *p* = 0.0071, *n* = 6–8). **e** Latency to feed in the novelty-suppressed feeding (NSF) test 4 days after i.p. and intra-mPFC injections (interaction, *F*_1,25_ = 6.81, *p* = 0.0151, *n* = 6–8). **f** Home cage feeding (HCF) just after the NSF (interaction, *F*_1,25_ = 0.356, *p* = 0.556, *n* = 6–8). **g** Schematic representation of mPFC infusion sites. Plates are from ref. [50]; 1.94, 1.78, 1.70, and 1.54 indicate distances (mm) from bregma. Data are expressed as means ± SEM. *p < 0.05, **p < 0.01, ***p < 0.001 (two-way ANOVA followed by Holm–Sidak’s post hoc test).

Previous studies have shown that signaling required for the synaptic and behavioral effects of ketamine occurs within the first 2 h [7], but ketamine induced long-lasting IGF-1 release in the mPFC (Fig. 1). Thus, we examined the effects of intra-mPFC infusion of the IGF-1 nAb 2 h after ketamine injection (Fig. 3a). Intra-mPFC infusion of IGF-1 nAb 2 h after ketamine injection completely blocked the antidepressant-like effects of ketamine in all three behavioral tests: the FST (Fig. 3b), FUST (Fig. 3d), and NSF test (Fig. 3e). There were no significant differences in LMA or HCF among groups (Fig. 3c, f). These results suggest that long-lasting IGF-1 release in the mPFC is required for the maintenance of the antidepressant-like effects of ketamine.

### Intra-mPFC co-infusion of IGF-1 nAb failed to block the antidepressant-like effects of intra-mPFC infusion of BDNF

**Fig. 4:**
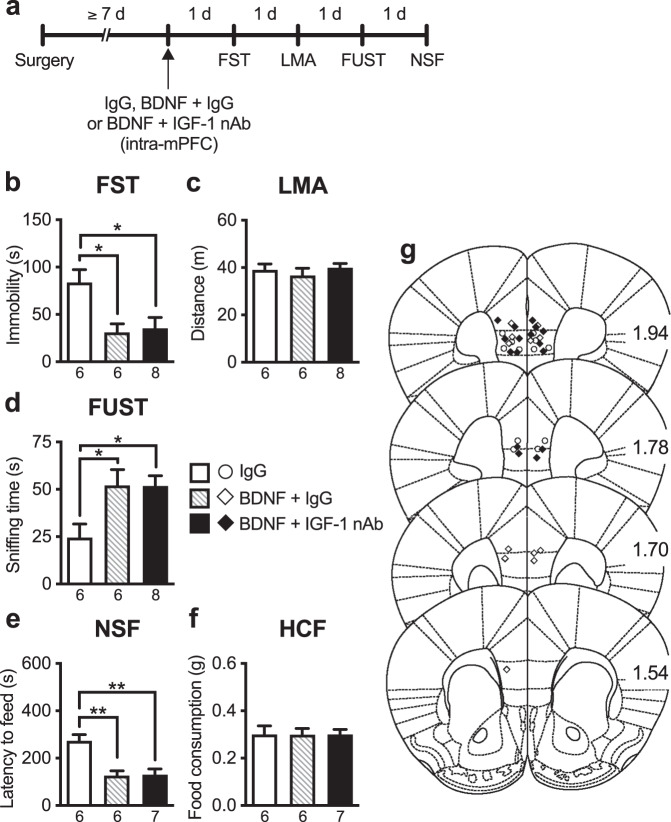
The antidepressant-like effects of intra-mPFC infusion of BDNF are not blocked by co-infusion of IGF-1 nAb. **a** Experimental timeline for behavioral testing after intra-mPFC infusion of control IgG (160 ng/side), BDNF (100 ng/side) plus control IgG, or BDNF plus IGF-1 nAb (160 ng/side). **b** Immobility time in the forced swim test (FST) 1 day after intra-mPFC infusion (*F*_2,17_ = 7.45, *p* = 0.0111, *n* = 6–8). **c** Locomotor activity (LMA) 2 days after intra-mPFC infusion (*F*_2,17_ = 0.514, *p* = 0.607, *n* = 6–8). **d** Time spent sniffing female urine in the female urine sniffing test (FUST) 3 days after intra-mPFC infusion (*F*_2,17_ = 5.14, *p* = 0.0179, *n* = 6–8). **e** Latency to feed in the novelty-suppressed feeding (NSF) test 4 days after intra-mPFC infusion (*F*_2,16_ = 12.4, *p* = 0.0006, *n* = 6–7). A BDNF + IGF-1 nAb-infused mouse was not subjected to the NSF because the mouse became lethargy, and exhibited ruffled fur and hunched posture as a result of overnight food deprivation. **f** Home cage feeding (HCF) just after the NSF (*F*_2,16_ = 2.98 × 10^−15^, *p* > 0.999, *n* = 6–7). **g** Schematic representation of mPFC infusion sites. Plates are from ref. [50]; 1.94, 1.78, 1.70, and 1.54 indicate distances (mm) from bregma. Data are expressed as means ± SEM. *p < 0.05, **p < 0.01 (one-way ANOVA followed by Tukey’s post hoc test).

Previous studies have shown that the rapid and sustained antidepressant-like effects of ketamine require BDNF release and signaling in the mPFC [9, 11, 13, 29], and we have demonstrated that a single intra-mPFC infusion of BDNF (100 ng/side) mimics the antidepressant-like actions of ketamine [21, 22, 30]. Here, to examine the role of IGF-1 in the antidepressant-like effects of intra-mPFC BDNF infusion, mice received intra-mPFC infusion of BDNF with or without IGF-1 nAb (160 ng/side; Fig. 4a). BDNF + control IgG-infused mice showed reduced immobility in the FST (Fig. 4b), increased sniffing of female urine in the FUST (Fig. 4d), and decreased latency to feed in the NSF (Fig. 4e). Intra-mPFC co-infusion of IGF-1 nAb failed to block these antidepressant-like effects of intra-mPFC BDNF infusion in the FST, FUST, and NSF test (Fig. 4b, d, e). There were no significant differences in LMA or HCF among groups (Fig. 4c, f). These results indicate that IGF-1 appears not to be a downstream mediator of the antidepressant-like actions of BDNF.

### Intra-mPFC co-infusion of BDNF nAb failed to block the antidepressant-like effects of intra-mPFC infusion of IGF-1

**Fig. 5:**
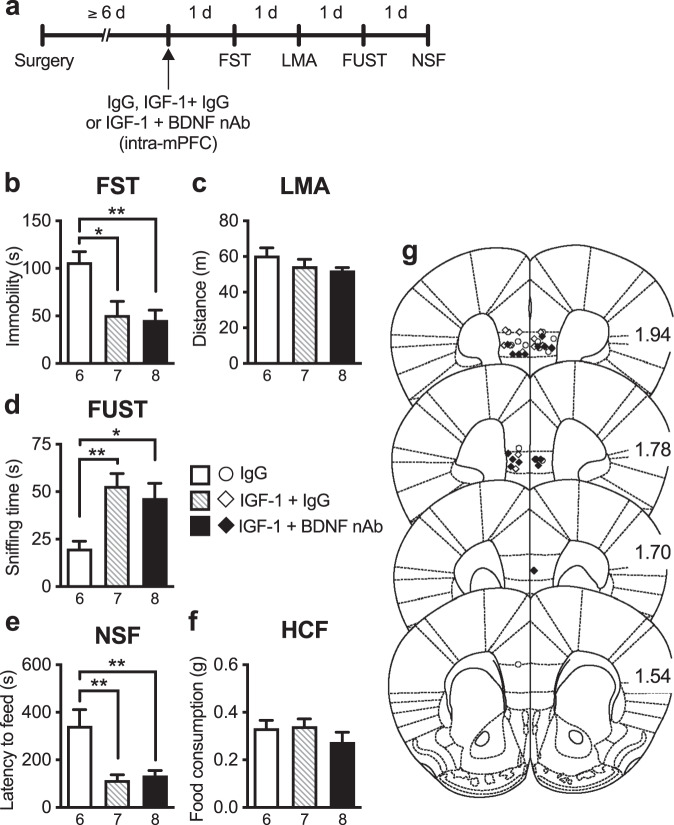
The antidepressant-like effects of intra-mPFC infusion of IGF-1 are not blocked by co-infusion of BDNF nAb. **a** Experimental timeline for behavioral testing after intra-mPFC infusion of control IgG (200 ng/side), IGF-1 (50 ng/side) plus control IgG, or IGF-1 plus BDNF nAb (200 ng/side). **b** Immobility time in the forced swim test (FST) 1 day after intra-mPFC infusion (*F*_2,18_ = 7.12, *p* = 0.0053, *n* = 6–8). **c** Locomotor activity (LMA) 2 days after intra-mPFC infusion (*F*_2,18_ = 1.66, *p* = 0.218, *n* = 6–8). **d** Time spent sniffing female urine in the female urine sniffing test (FUST) 3 days after intra-mPFC infusion (*F*_2,18_ = 6.15, *p* = 0.0092, *n* = 6–8). **e** Latency to feed in the novelty-suppressed feeding (NSF) test 4 days after intra-mPFC infusion (*F*_2,18_ = 10.12, *p* = 0.0011, *n* = 6–8). **f** Home cage feeding (HCF) just after the NSF (*F*_2,18_ = 1.10, *p* = 0.354, *n* = 6–8). **g** Schematic representation of mPFC infusion sites. Plates are from ref. [50]; 1.94, 1.78, 1.70, and 1.54 indicate distances (mm) from bregma. Data are expressed as means ± SEM. *p < 0.05, **p < 0.01 (one-way ANOVA followed by Tukey’s post hoc test).

We also examined whether the antidepressant-like effects of intra-mPFC IGF-1 infusion (50 ng/side) were blocked by co-infusion of BDNF nAb (200 ng/side; Fig. 5a). IGF-1 + control IgG-infused mice displayed reduced immobility in the FST (Fig. 5b), increased sniffing of female urine in the FUST (Fig. 5d), and decreased latency to feed in the NSF (Fig. 5e). Intra-mPFC co-infusion of BDNF nAb failed to block these antidepressant-like actions of intra-mPFC IGF-1 infusion in the FST, FUST, and NSF test (Fig. 5b, d, e). There were no significant differences in LMA or HCF among groups (Fig. 5c, f). These results, in combination with those in Fig. 4, suggest that BDNF and IGF-1 contribute independently of each other to the antidepressant-like effects of ketamine.

### Intra-mPFC infusion of IGF-1 nAb at 2 h post-ketamine blocks the antidepressant-like effects of ketamine in LPS-induced depression model mice

**Fig. 6:**
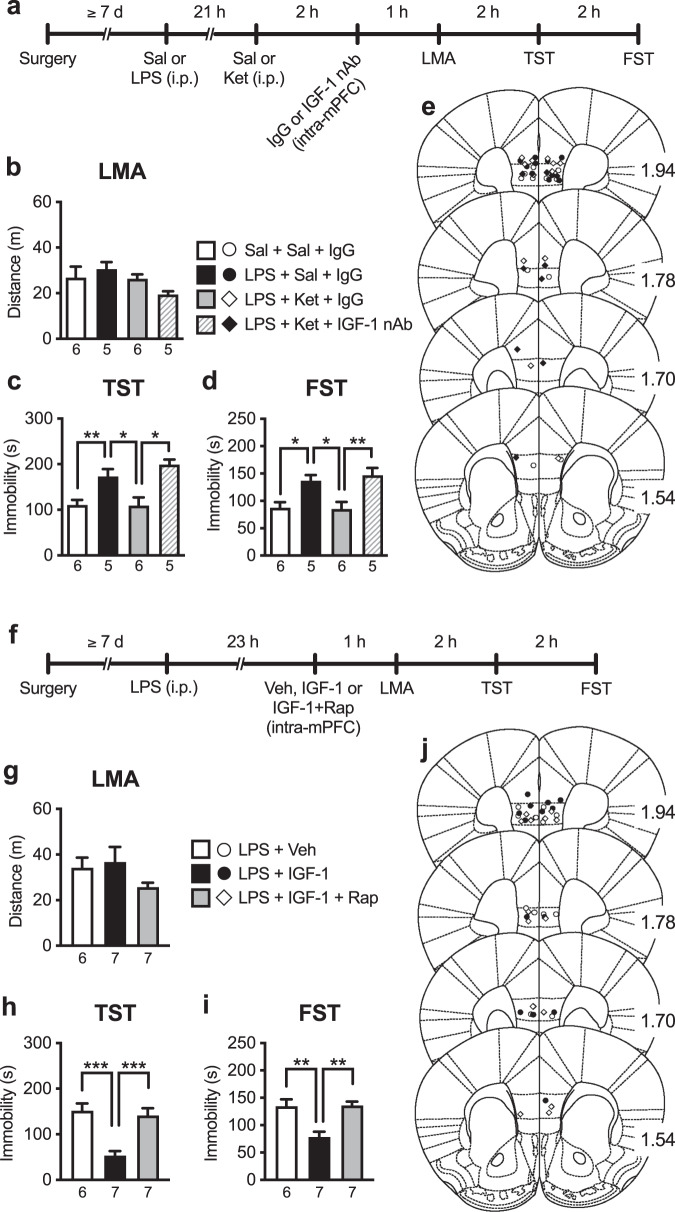
Intra-mPFC infusion of IGF-1 nAb at 2 h post-ketamine blocks the antidepressant-like effects of ketamine in LPS-induced depression model mice: involvement of mTORC1 signaling in the antidepressant-like actions of IGF-1. **a** Experimental timeline for LPS challenge (0.8 mg/kg, i.p.), i.p. injection of either saline or ketamine (10 mg/kg), intra-mPFC infusion of either control IgG (160 ng/side) or IGF-1 nAb (160 ng/side), and behavioral testing. **b** Locomotor activity (LMA) 24 h after LPS challenge (*F*_3,18_ = 1.92, *p* = 0.163, *n* = 5–6). **b** Immobility time in the tail suspension test (TST) 26 h after LPS challenge (*F*_3,18_ = 9.43, *p* = 0.0006, *n* = 5–6). **c** Immobility time in the forced swim test (FST) 28 h after LPS challenge (*F*_3,18_ = 7.45, *p* = 0.0019, *n* = 5–6). **e** Schematic representation of mPFC infusion sites. Plates are from ref. [50]; 1.94, 1.78, 1.70, and 1.54 indicate distances (mm) from bregma. **f** Experimental timeline for LPS challenge, intra-mPFC infusion of vehicle (10% DMSO/0.09% BSA/PBS), IGF-1 (50 ng/side) or IGF-1 plus rapamycin (0.01 nmol/side), and behavioral testing. **g** LMA 24 h after LPS challenge (*F*_2,17_ = 1.55, *p* = 0.242, *n* = 6–7). **h** Immobility time in the TST 26 h after LPS challenge (*F*_2,17_ = 14.9, *p* = 0.0002, *n* = 6–7). **i** Immobility time in the FST 28 h after LPS challenge (*F*_2,17_ = 11.6, *p* = 0.0007, *n* = 6–7). **j** Schematic representation of mPFC infusion sites. Plates are from ref. [50]; 1.94, 1.78, 1.70, and 1.54 indicate distances (mm) from bregma. Data are expressed as means ± SEM. *p < 0.05, **p < 0.01 (one-way ANOVA followed by Tukey’s post hoc test).

To further confirm the role of IGF-1 in the mPFC in the antidepressant-like actions of ketamine, we examined whether intra-mPFC infusion of IGF-1 nAb 2 h after ketamine injection can reverse depression-like behaviors induced by LPS challenge in the TST and FST (Fig. 6a). LPS challenge and these treatments did not significantly affect LMA (Fig. 6b). LPS challenge in saline + control IgG-treated mice (LPS + Sal + IgG group) significantly increased immobility in both the TST (Fig. 6c) and FST (Fig. 6d), and these depression-like behaviors were alleviated by ketamine in control IgG-infused mice (LPS + Ket + IgG group; Fig. 6c, d). These antidepressant-like effects of ketamine were completely blocked by intra-mPFC infusion of IGF-1 nAb (LPS + Ket + IGF-1 nAb group; Fig. 6c, d). These results suggest that IGF-1 release in the mPFC mediates the antidepressant-like actions of ketamine in the LPS-induced depression model mice.

### Intra-mPFC co-infusion of mTORC1 inhibitor blocks the antidepressant-like effects of intra-mPFC infusion of IGF-1 in LPS-induced depression model mice

Previous studies have demonstrated that mTORC1 signaling in the mPFC is essential for the antidepressant-like actions of ketamine [7–9, 13] and that IGF-1 was reported to activate mTORC1 signaling in cultured neurons [31]. Thus, to examine the role of mTORC1 in the antidepressant-like actions of intra-mPFC IGF-1 infusion (50 ng/side), mice received intra-mPFC infusion of IGF-1 with or without the mTORC1 inhibitor rapamycin (0.01 nmol/side) 23 h after LPS challenge (Fig. 6f). There was no significant difference in LMA among groups (Fig. 6g). IGF-1-infused mice (LPS + IGF-1 group) significantly decreased immobility in both the TST and FST compared with vehicle (10% DMSO/0.09% BSA/PBS)-infused control mice (LPS + Veh group), and these antidepressant-like effects of IGF-1 were completely blocked by intra-mPFC co-infusion of rapamycin (LPS + IGF-1 + Rap group; Fig. 6h, i). These results suggest that mTORC1 activation is essential for the antidepressant-like actions of intra-mPFC IGF-1 infusion.

## Discussion

In this study, in vivo microdialysis analysis showed that ketamine induced persistent IGF-1 release in the mPFC. Intra-mPFC sequestration of extracellular IGF-1 by nAb 15 min before or 2 h after ketamine administration was sufficient to block the antidepressant-like effects of ketamine in three different behavioral paradigms, including models of behavioral despair (FST), motivation/reward (FUST), and anxiety (NSF). These results suggest that IGF-1 release in the mPFC is necessary for the induction and maintenance of the rapid and sustained antidepressant-like effects of ketamine. Moreover, the results demonstrated that intra-mPFC infusion of IGF-1 nAb 2 h after ketamine injection blocked the antidepressant-like actions of this drug in the murine LPS-induced depression model. Although further studies will be required to examine the role of IGF-1 release within the mPFC in the antidepressant-like effects of ketamine using chronic stress models of depression, including chronic unpredictable stress (CUS) and chronic social defeat stress models [8, 32], to our knowledge, this is the first study to suggest that IGF-1 release in the mPFC mediates the antidepressant-like actions of ketamine.

Our in vivo microdialysis analysis showed that ketamine administration (10 and 30 mg/kg, i.p.) persistently increased extracellular IGF-1 levels in the mPFC, while serum IGF-1 levels remained unchanged 3 h after administration of ketamine (10 mg/kg). Given that the half-life of ketamine in mouse plasma is approximately 0.5 h [33], our present findings suggest that ketamine-induced plastic changes in the mPFC cause persistent IGF-1 release in naïve mice. Further studies are needed to examine whether ketamine can induce persistent IGF-1 release in the mPFC of rodent depression models such as the LPS-challenged animals in which IGF-1 levels in the frontal cortex are decreased [18, 34].

Previous studies have reported that several types of neurons including olfactory bulb neurons, midbrain dopaminergic neurons, and hippocampal CA1 pyramidal neurons, release IGF-1 in an activity-dependent manner [35–37]. Both IGF-1 and IGF-1 receptors are expressed in the frontal cortex, and its paracrine action has also been suggested [38, 39]. Thus, we hypothesize that ketamine induces activity-dependent release of IGF-1 from mPFC pyramidal neurons, as well as BDNF [9, 13]; however, it is technically difficult to measure extracellular BDNF levels in vivo by our microdialysis procedure. As IGF-1 is also reportedly released from astrocytes and microglia [40], we cannot exclude the possibility that IGF-1 derived from glial cells contributes to the antidepressant-like actions of ketamine. Thus, future studies using cell type-specific deletion of IGF-1 are required to determine the source(s) of IGF-1 responsible for the actions of ketamine.

BDNF is a key mediator of the antidepressant-like effects of ketamine and its enantiomers [(*R*)-ketamine and (*S*)-ketamine] [9, 11, 13, 29, 41]. The present study demonstrated that the antidepressant-like effects of BDNF persisted after intra-mPFC co-infusion of BDNF and IGF-1 nAb. Furthermore, the antidepressant-like effects of IGF-1 persisted after intra-mPFC co-infusion of IGF-1 and BDNF nAb. Altogether, these findings suggest that BDNF and IGF-1 mediate the antidepressant-like effects of ketamine independently. In contrast, previous studies suggest an interaction between IGF-1 and BDNF. These studies showed that exogenously administered IGF-1 reportedly induces BDNF expression in the brain [17, 42], and that pretreatment of IGF-1 enhances BDNF signaling in BDNF-treated primary cortical neurons [43]. The reason for this discrepancy is currently unknown. One possible explanation is that BDNF and IGF-1 may produce antidepressant-like effects before the co-infused nAbs can sufficiently block the actions of the neurotrophic factors. In future studies, it would be interesting to address this issue and determine the specific roles of these neurotrophic factors in the antidepressant-like actions of ketamine and its enantiomers.

It has been reported that ketamine rapidly activates mTORC1 signaling within 1 h, increases synaptic proteins [postsynaptic density (PSD)−95, synapsin-1, and GluA1 subunit of AMPA receptor] in synaptoneurosome preparations of the prefrontal cortex in an mTORC1-dependent manner as early as 2 h after administration. Additionally, ketamine enhances serotonin- and hypocretin-induced excitatory postsynaptic current responses in mPFC layer V pyramidal neurons and increases dendritic spine density in the mPFC within 24 h of treatment [7, 44, 45]. Moreover, these neuroplastic changes in the mPFC are associated with the antidepressant-like effects of ketamine [9].

Interestingly, IGF-1 also activates mTORC1 signaling in cultured hippocampal neurons [31]. Consistent with this finding, the current study demonstrated that intra-mPFC IGF-1 infusion produces antidepressant-like effects in the LPS-induced depression model mice via mTORC1 activation. Furthermore, IGF-1 increases cell surface GluA1 protein levels in the mPFC [15], and exogenous IGF-1 has also been reported to increase excitability and potentiate synaptic transmission in mPFC layer V pyramidal neurons [15, 46]. Additionally, the frontoparietal cortex of IGF-1 null mice have reduced dendritic spine density in layers II/III and V pyramidal neurons [47], while IGF-1 treatment rescued decreased PSD-95 protein levels and dendritic spine density in the somatosensory cortex of a mouse model of cyclin-dependent kinase-like 5 deficiency disorder [48]. Moreover, Burgdorf et al. [15] reported that a single intravenous injection of recombinant IGF-1 produces long-lasting antidepressant-like effects in rats exposed to CUS and that a single intra-mPFC infusion of IGF-1 exerts antidepressant-like effects in the FST, similar to the current results. Altogether, these findings suggest that endogenous IGF-1 in the mPFC could induce the neuroplastic effects of ketamine, possibly via mTORC1 activation, which may be the underlying mechanism of the induction of the antidepressant-like effects of this drug. The current results also demonstrated that the antidepressant-like actions of ketamine are completely blocked by intra-mPFC IGF-1 nAb infusion even 2 h after the treatment. At this time, the signaling required for the induction of behavioral and synaptic actions of ketamine has already occurred [7]. Based on these findings, we hypothesize that the ketamine-induced sustained increase in extracellular IGF-1 levels may contribute to increasing long-term survival and/or stability of pre-existing and newly formed spines in mPFC pyramidal neurons, resulting in the sustained antidepressant-like actions of ketamine. This hypothesis should be tested in future studies.

In summary, the present findings demonstrate that IGF-1 signaling in the mPFC is essential for the rapid and sustained antidepressant effects of ketamine, but not BDNF. IGF-1 has been approved in many countries for the treatment of dwarfism and reportedly produces antidepressant and anxiolytic effects in obese postmenopausal women [49]. Thus, IGF-1 and its receptors may be potential targets for the development of novel rapid-acting antidepressants. However, the present study has some limitations. Although women are diagnosed with MDD roughly twice as often as men [1], this study was performed entirely in male mice. Moreover, the present study did not examine the role of IGF-1 in the antidepressant-like actions of ketamine enantiomers. It will be important to address these issues in future studies.
